# Estrogen activates pyruvate kinase M2 and increases the growth of TSC2-deficient cells

**DOI:** 10.1371/journal.pone.0228894

**Published:** 2020-02-20

**Authors:** Yiyang Lu, Xiaolei Liu, Erik Zhang, Elizabeth J. Kopras, Eric P. Smith, Aristotelis Astreinidis, Chenggang Li, Yuet-Kin Leung, Shuk-Mei Ho, Jane J. Yu

**Affiliations:** 1 University of Cincinnati College of Medicine, Department of Internal Medicine, Cincinnati, OH, United States of America; 2 Division of Pediatric Nephrology, Department of Pediatrics, College of Medicine, University of Tennessee Health Sciences Center and Tuberous Sclerosis Complex Center of Excellence, Le Bonheur Children’s Hospital, Memphis, TN, United States of America; 3 State Key Laboratory of Medicinal Chemical Biology and College of Pharmacy, Nankai University, Tianjin, China; 4 College of Medicine Department of Pharmacology and Toxicology, the University of Arkansas for Medical Science (UAMS), Little Rock, AR, United States of America; Florida International University, UNITED STATES

## Abstract

Lymphangioleiomyomatosis (LAM) is a devastating lung disease caused by inactivating gene mutations in either *TSC1* or *TSC2* that result in hyperactivation of the mechanistic target of rapamycin complex 1 (mTORC1). As LAM occurs predominantly in women during their reproductive age and is exacerbated by pregnancy, the female hormonal environment, and in particular estrogen, is implicated in LAM pathogenesis and progression. However, detailed underlying molecular mechanisms are not well understood. In this study, utilizing human pulmonary LAM specimens and cell culture models of TSC2-deficient LAM patient-derived and rat uterine leiomyoma-derived cells, we tested the hypothesis that estrogen promotes the growth of mTORC1-hyperactive cells through pyruvate kinase M2 (PKM2). Estrogen increased the phosphorylation of PKM2 at Ser37 and induced the nuclear translocation of phospho-PKM2. The estrogen receptor antagonist Faslodex reversed these effects. Restoration of TSC2 inhibited the phosphorylation of PKM2 in an mTORC1 inhibitor-insensitive manner. Finally, accumulation of phosphorylated PKM2 was evident in pulmonary nodule from LAM patients. Together, our data suggest that female predominance of LAM might be at least in part attributed to estrogen stimulation of PKM2-mediated cellular metabolic alterations. Targeting metabolic regulators of PKM2 might have therapeutic benefits for women with LAM and other female-specific neoplasms.

## Introduction

Lymphangioleiomyomatosis (LAM) is a disease that develops almost exclusively in females of reproductive age and predominantly involves the lungs. Although the genetic basis is known, specifically mutations in either tuberous sclerosis 1 (*TSC1*) or the tuberous sclerosis 2 (*TSC2*) genes, the pathophysiology is poorly understood. It is hypothesized that smooth-muscle-like cells of uncertain origin, but likely the uterus, and with inactivating mutations in *TSC1* or *TSC2* genes disseminate via the lymphatics primarily to the lungs followed by proliferation and progressive cystic destruction of lung parenchyma. Cells within the cystic LAM lesions produce matrix metalloproteases and growth factors, such as vascular endothelial growth factor (VEGF)-D, which contribute to lung remodeling [1]. Although the exact mechanisms for the strong female predominance remain elusive, sex hormone dependence is clear as symptoms are exacerbated during pregnancy [2–4] and sex steroid hormone receptors are present in LAM nodules [5–8].

A possible insight into the mechanism of action of estrogen in LAM derives from studies on energy, lipid and substrate metabolism regulated by the mechanistic target of rapamycin complex 1 (mTORC1). Cells with mutations in the TSC genes have increased expression of genes encoding the enzymes for lipid and sterol biosynthesis, glycolysis, and the pentose phosphate pathway [9], all pathways critical for cell growth. Hyperactive mTORC1 stimulates pyruvate kinase muscle isozyme M2 (PKM2) [10], the rate limiting glycolytic enzyme, which catalyzes the final step in glycolysis. PKM2 plays a central role in the metabolic reprogramming of cancer cells, cell cycle progression, and gene transcription [11]. PKM2-stimulated glycolysis contributes to the development of tumors caused by hyperactive mTORC1 [10], and, in part, this is mediated through induction of HIF-1α expression [10]. The phosphorylation of PKM2 at Ser37, by extracellular signal-regulated kinase (ERK), promotes PKM2 translocation to the nucleus, where it affects regulation of genes involved in glycolysis [12, 13]. Our previous studies showed that estrogen treatment is associated with further elevation of pentose phosphate pathway intermediates and the proliferation of TSC2-deficient cells [14]. Specifically, estrogen treatment increased glucose uptake and the levels of pentose phosphate pathway signatures including glucose-6-phosphate, fructose-6-phosphate, ribose, ribose-5-phosphate and ribulose-5-phosphate, in TSC2-deficient ELT3 and 621–101 cells [14]. Moreover, estrogen treatment significantly induced Erk1/2 phosphorylation in Tsc2-deficient ELT3 cells [14–18].

In this study, we addressed further the mechanism of estrogen action on PKM2. We report that estrogen increases the phosphorylation of PKM2 at Ser37 and induces the nuclear translocation of phospho-PKM2. Treatment with the estrogen receptor antagonist Faslodex blocks estrogen-induced nuclear translocation of phospho-PKM2. Re-expression of TSC2 decreases the protein levels and phosphorylation of PKM2 in an mTORC1 inhibitor-insensitive manner. Accumulation of phosphorylated PKM2 was evident in pulmonary nodule cells, from TSC/LAM patients. Collectively, our study reveals that PKM2-mediated glucose metabolic reprogramming may contribute to estrogen-dependent LAM cell growth and the pathogenesis of LAM. Thus, targeting metabolic regulators of PKM2 might have therapeutic benefits for women with LAM and other female-specific mTORC1-hyperactive neoplasms.

## Materials and methods

### Cell culture and reagents

MCF7 and A589 cells were obtained from ATCC and cultured in DMEM supplemented with 10%FBS. ELT3 cells were provided by Dr. C. Walker [19, 20]. ELT3-V3 (Tsc2-), ELT3-T3 (TSC2+) [21], 621–101 and 621–103 cells were provided by Dr. E.P. Henske [22]. Cells were cultured in IIA complete medium supplemented with sodium selenite 5 × 10^−8^ mol/L, insulin 25 μg/mL, hydrocortisone 2 × 10^−7^ mol/L, transferrin 10 μg/mL, T3 10^−9^ mol/L, vasopressin 10 μU/mL, cholesterol 10^−8^ mol/L, ferrous sulfate 1.6 × 10^−6^ mol/L, EGF 10 ng/mL, and 10% FBS. Advanced DMEM/F-12 (Thermo Fisher Scientific) was used as glucose-free basal medium. 17-β-estradiol (E_2_) (10 nM, Sigma-Aldrich), Faslodex (Fulvestrant, 10 μM, Sigma-Aldrich), PD98059 (50 μM, Sigma-Aldrich), and Rapamycin (10 nM, Enzo Life Sciences, Inc) were used.

### Immunofluorescence and immunohistochemistry analysis

Cells seeded in chambers of Millicell EZ slides (Millipore) were fixed and incubated with primary antibody against phospho-PKM2 [Ser37] using a 1:100 dilution, Alexa Fluor dye-conjugated secondary antibodies and SlowFade® Gold reagent for mounting were from Invitrogen Life Science Technologies. Immunohistochemistry was performed on paraffin-embedded 10 μm sections using antibodies against Phospho-PKM2 [Ser37], phospho-S6 [Ser235/236], and α-smooth muscle actin. Images were captured using Olympus CellSens imaging software.

### Lentiviral infection

shRNA lentiviral constructs were obtained from Lenti-shRNA Library Core at the Cincinnati Children’s Hospital Medical Center. Envelope pMD2.G and packaging psPAX2 were co-transfected into HEK293T cells using Lipofectamine 2000 transfection reagent (Invitrogen). Lentiviral particles were collected 24 hours post transfection to infect 621–101 cells in the presence of 8 mg/mL polybrene. Stable clones were selected with 10 μg/mL puromycin.

### Nucleofection

The plasmids of pcDNA3.1(+)-TSC2 or empty vector pcDNA3.1(+) were transfected in 621–101 cells using 4D-NucleofectorTM X Kit L (#V4XC-2024, Lonza). 1x10^6^ cells were suspended in 100 μl nucleofection solution containing plasmids, and then subjected to electrical pulse in Lonza 4D–NucleofectorTM Core/X Unit (Lonza).

### Measurement of cell growth

Cells were seeded in 96-well plates, treated with E_2_ (10 nM) or vehicle for the indicated times and in the indicated medium, followed by crystal violet staining and measurement in a microplate reader (BioTek).

### Quantitative real-time PCR

Total RNA was extracted using the RNeasy mini kit (Qiagen). cDNA was synthesized from 2 μg of total RNA using a high-capacity cDNA reverse transcription kit (Applied Biosystems) with random primers [23]. Gene expression was quantified using SYBR green real-time PCR Master Mixes kit (Life Technologies) in the Applied Biosystems Real-Time PCR System and normalized to β-actin or tubulin. The human primers used were:

ESR1 (ERα): Forward: 5’-GCTTACTGACCAACCTGGCAGA-3’.

Reverse: 5’-GGATCTCTAGCCAGGCACATTC-3’.

ESR2 (ERβ): Forward: 5’-AGCTGGGCCAAGAAGATTCC-3’.

Reverse: 5’-TGCCAGGAGCATGTCAAAGA-3’.

β-actin: Forward: 5’-CACCATTGGCAATGAGCGGTTC-3’.

Reverse: 5’-AGGTCTTTGCGGATGTCCACGT-3’.

The rat primers used were:

Esr1 (ERα): Forward: 5’-AGGCTGCAAGGCTTTCTT-3’.

Reverse: 5’-CAACTCTTCCTCCGGTTCTTATC-3’.

Esr2 (ERβ): Forward: 5’-ATGTACCCCTTGGCTTCTGC-3’.

Reverse: 5’-TCTGTAGTCTGTCCGCCTCA-3’.

Tubulin: Forward: 5’-GAGGAGATGACTCCTTCAACACC-3’.

Reverse: 5’-TGATGAGCTGCTCAGGGTGGAA-3’.

### Subcellular fractionation and western blotting

Cytoplasmic and nuclear fractions were isolated using the Subcellular Protein Fractionation Kit (Thermo Scientific). Anti-Phospho-PKM2 [Ser37] was from Signalway Antibody. Anti-β-Actin (AC-15) was from Sigma. Antibodies to PKM2, Tuberin (TSC2), Raptor, Rictor, Phospho-ERK1/2, Phospho-Akt [Ser473], phospho-S6 [Ser235/236], and phospho-S6K [Thr389] were from Cell Signaling. Anti-SMA was from Abcam Antibodies to NUPL1 and S6 were from Santa Cruz Biotechnology.

### Human samples

Pulmonary LAM tissues from TSC/LAM patients were obtained from the National Disease Research Interchange (NDRI).

### Statistical analyses

Statistical analyses were performed using two-sided Student’s *t*-test when comparing two groups. Results are presented as means ± SEM.

## Results

### Estrogen promotes the growth of TSC2-deficient cells in a glucose-dependent manner *in vitro*

**Fig 1:**
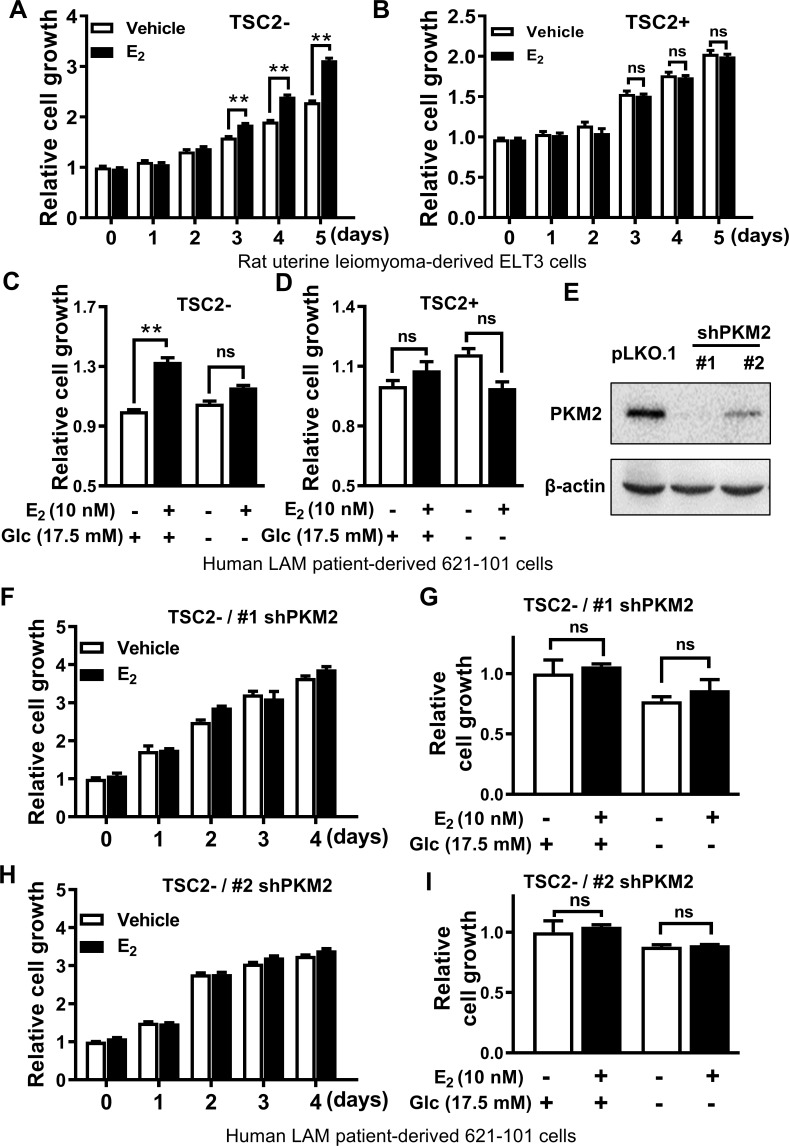
Estrogen promotes the growth of TSC2-deficient cells via PKM2 in a glucose-dependent manner. Cell growth was compared in **(A)** TSC2-deficient (Tsc2-) ELT3 cells and **(B)** TSC2-add back (Tsc2+) ELT3 cells, at the indicated time points over a range of 5 days after E_2_ treatment. Data were normalized by non-treatment control (n = 8). Cell growth was measured by crystal violet assay in TSC2-deficient 621–101 cells **(C)** and TSC2-addback 621–103 cells **(D)** after 24 h of E_2_ treatment and culture in glucose-free (Glc -) or glucose-rich (Glc 17.5 mM) medium (n = 8). **(E)** Immunoblot analysis of PKM2 in 621–101 cells infected with lentiviral particles of shRNA-PKM2 (#1 and #2) targeting different regions within the same gene or of empty vector pLKO.1 as control. **(F, H)** shRNA-PKM2 (#1 and #2) 621–101 cells were treated with E_2_ (10 nM) or vehicle over a range of 4 days. Cell growth was measured by crystal violet assay; data were normalized to vehicle control at day 0 (n = 8). **(G, I)** shRNA-PKM2 (#1 and #2) 621–101 cells were cultured in glucose-rich (Glc 17.5 mM) or glucose-deprived (Glc 0 mM), and then treated with 10 nM E_2_ or vehicle for 24 hours (n = 8). Cell growth was measured using crystal violet staining; data were normalized to the vehicle treatment and glucose-rich group. Data are represented as mean ± SEM, **p<0.01, ns: not significant, two-sided Student’s t-test.

To elucidate pro-survival mechanisms regulated by estrogen in LAM cells [17, 19, 24], we used the well-established, estrogen-responsive Tsc2-deficient rat ELT3 cell line, which was engineered to express an empty vector (TSC2-) or TSC2 add-back (TSC2+) [21]. Consistent with our findings [17] and those of others [15, 19], estrogen modestly promoted the growth of ELT3 (TSC2-) cells by 12%, 25%, and to 35% in a time-dependent manner from day 3 to day 5, respectively (p < 0.01; **Fig 1A**). However, estrogen treatment had no measurable effect on the growth of ELT3 cells expressing TSC2 (TSC2+) (**Fig 1B**). We also tested estrogen responsiveness in LAM patient-derived TSC2-deficient 621–101 cells that exhibit constitutively active mTORC1, and in TSC2-expressing 621–103 cells. Consistently, crystal violet assay showed that estrogen significantly increased cell number by approximately 33% (p < 0.01) but only in glucose-rich medium (**Fig 1C**). In contrast, and consistent with the results shown in Fig 1B, estrogen treatment did not affect the growth of TSC2-addback 621–103 (TSC2+) cells regardless of glucose concentrations (**Fig 1D**). These data indicate that estrogen selectively promotes the growth of TSC2- cells in a glucose-dependent manner.

To examine the effect of PKM2 knockdown on glucose- and estrogen-dependent growth of TSC2-null cells, we depleted *PKM2* using two independent shRNAs in 621–101 cells. Immunoblot analysis showed that the protein levels of PKM2 were reduced by 95.1% (PKM2 shRNA#1) and 80.4% (PKM2-shRNA#2), relative to pLKO.1 vector control, respectively (**Fig 1E**). Importantly, E_2_ treatment for 4 days did not stimulate the growth of 621-101-PKM2-shRNA#1 **(Fig 1F)** or 621-101-PKM2-shRNA#2, relative to vehicle control, respectively **(Fig 1H)**. Moreover, E_2_ treatment did not affect the growth of 621–101 cells depleted with PKM2 (shRNA#1 and #2) in glucose-rich (Glc 17.5 mM) and glucose-free conditions **(Fig 1G and 1I)**. Together, our data strongly support a specific role for PKM2 in glucose- and estrogen-dependent growth of TSC2-null cells.

### Estrogen regulates PKM2 phosphorylation in TSC2-deficient cells *in vitro*

**Fig 2:**
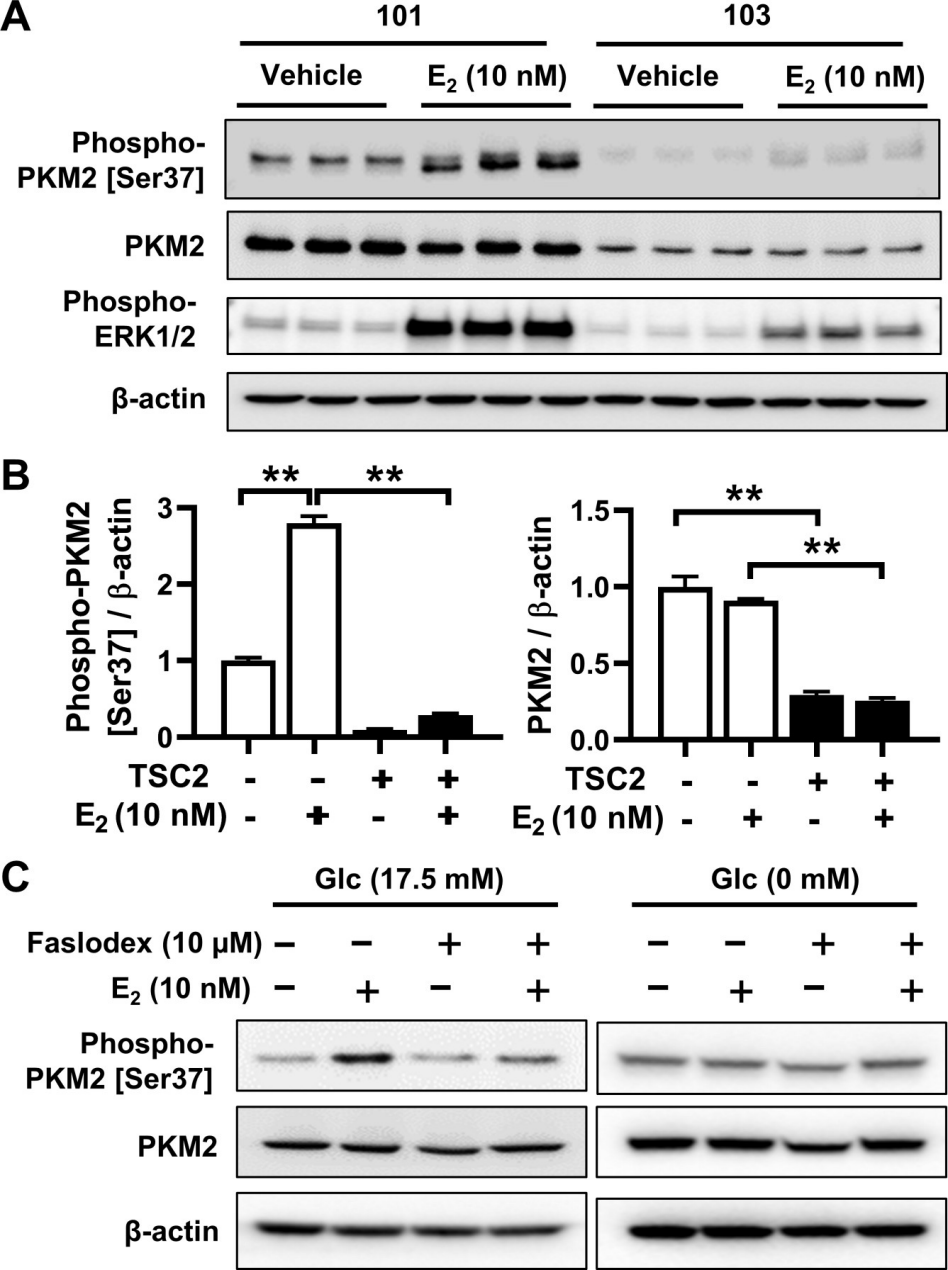
Estrogen induces PKM2 phosphorylation. **(A)** 621–101 and 621–103 cells in triplicate after E_2_ (10 nM) treatment for 2 hours. Immunoblot analysis of phospho-PKM2 [Ser37], PKM2 and Phospho-ERK1/2 [Thr202/Tyr204]. **(B)** Densitometry analysis of phospho-PKM2 [Ser37] and PKM2 normalized to β-actin, respectively (n = 3). Results are representative of three experiments, and data are represented as mean ± SEM. **p<0.01, two-sided Student’s t-test. **(C)** 621–101 cells were treated with vehicle, E_2_ (10 nM), Faslodex (10 μM), or E_2_ (10 nM) plus Faslodex (10 μM) for 24 hours in glucose-rich (Glc 17.5 mM) or glucose-free medium (Glc 0 nM), followed by immunoblot analysis of phospho-PKM2 [Ser37] and PKM2. β-actin as a loading control.

Although PKM2-stimulated glycolysis contributes to the development of tumors caused by hyperactive mTORC1 [10], the impact of PKM2 on HIF-1α transcription, and ultimately cell proliferation, requires phosphorylation of PKM2 at Ser37 by extracellular signal-regulated kinase (ERK1/2). Phosphorylated-PKM2 translocates from the cytoplasm to the nucleus and regulates the expression of genes involved in glycolysis [12, 13]. We speculated that estrogen induces the phosphorylation of PKM2 at Ser37 through activating the ERK1/2 pathway. Estrogen treatment for 2 hours markedly increased the levels of phospho-PMK2 [Ser37], but had no effect on the protein levels of PKM2, relative to the vehicle control, in 621–101 cells (**Fig 2A**). Quantitative densitometry of three biological replicates showed the significant increase of 2.8-fold for phospho-PMK2, whereas no significant difference for PKM2 (**Fig 2B**). Importantly, E_2_-induced PKM2 phosphorylation was concomitant with robust phosphorylation of ERK1/2 in 621–101 cells (**Fig 2A**). Collectively, these results indicate that estrogen-induced PKM2 phosphorylation is unequivocal and, moreover, is associated with ERK1/2 activation in 621–101 cells.

Next, we assessed the effect of TSC2 expression on PKM2 phosphorylation. We found that the basal level of phospho-PKM2 [Ser37] was significantly lower in TSC2-corrected 621–103 cells relative to that in TSC2-null 621–101 cells (**Fig 2A and 2B**). Moreover, the level of E_2_-induced PKM2 phosphorylation was substantially higher in 621–101 (TSC2-) relative to that in 621–103 (TSC2+) cells. Our data indicate that TSC2 negatively regulates PKM2 phosphorylation and suppresses E_2_-induced PKM2 phosphorylation in LAM-derived cells.

To further investigate the effect of estrogen inhibition on activation of PKM2, we treated 621–101 TSC2-null cells with Faslodex, a pure estrogen receptor antagonist, for 24 hours. Faslodex treatment markedly decreased E_2_-induced PKM2 phosphorylation at Ser37, although the protein levels of PKM2 were not significantly affected (**Fig 2C**). To examine the effect of glucose and estrogen on PKM2 phosphorylation, we cultured 621–101 cells in glucose-rich or glucose-free conditions for 24 hours, and then treated cells with 10 nM E_2_ for 2 hours. Under glucose-rich conditions (Glc 17.5 mM), E_2_ stimulation largely increased the level of phospho-PKM2 [Ser37] relative to vehicle control (**Fig 2C, left panel**). Moreover, treatment with faslodex, an estrogen receptor alpha (ERα) antagonist, completely prevented E_2_-induced PKM2 phosphorylation (**Fig 2C, left panel**). Furthermore, E_2_ stimulation did not increase the levels of phospho-PKM2 in 621–101 cells under glucose-free (Glc 0 nM) conditions (**Fig 2C, right panel**), further supporting the important impact of glucose and estrogen on PKM2 phosphorylation in TSC2-null LAM patient-derived cells.

### Estrogen induces nuclear localization of phosphorylated PKM2

**Fig 3:**
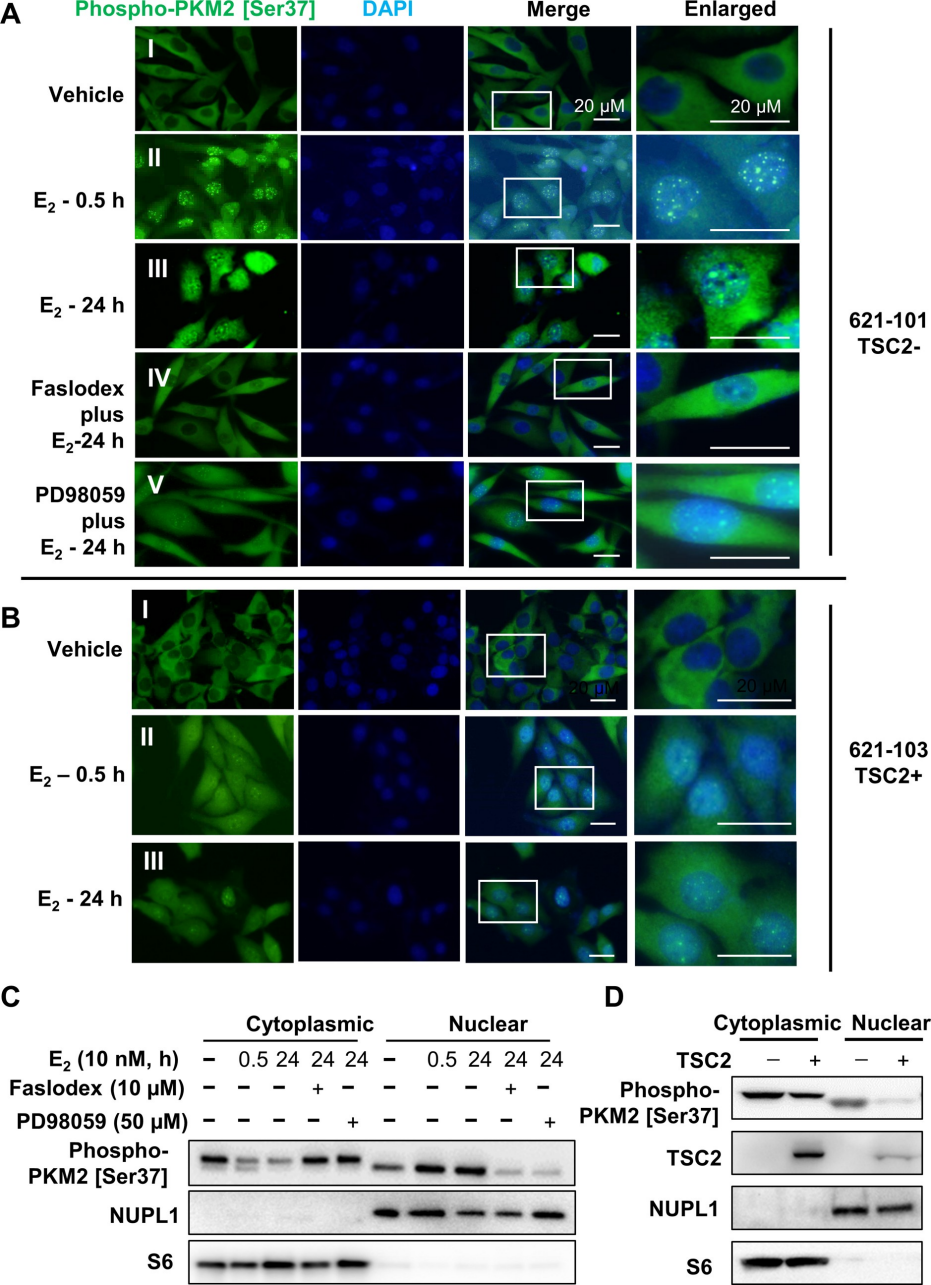
Estrogen induces nuclear translocation of phospho-PKM2 [S37] in a TSC2-dependent manner. **(A)** Immunofluorescence staining of phospho-PKM2 [Ser37] in 621–101 cells with the treatment of (I) Vehicle, (II) E_2_ (10 nM) for 0.5 hours, (III) E_2_ (10 nM) for 24 hours, and combination of E_2_ (10 nM) with (IV) Faslodex (10 μM) or (V) PD98059 (50 μM) for 24 hours. **(B)** Immunofluorescence staining of phospho-PKM2 [Ser37] in TSC2-reexpressing 621–103 (TSC2+) cells treated with (I) Vehicle, (II) E_2_ (10 nM) for 0.5 hours, and (III) E_2_ (10 nM) for 24 hours. Nuclei were stained with DAPI. Scale bar represents 20 μm. **(C)** Immunoblot analysis of phospho-PKM2 [Ser37], NUPL1 and S6 in cytoplasmic and nuclear fractions isolated from 621–101 cells in the same treatment as (A). **(D)** Immunoblot analysis of phospho-PKM2 [Ser37], TSC2, NUPL1 and S6 in cytoplasmic and nuclear fractions isolated from 621–101 (TSC2-) and 621–103 (TSC2+) cells.

Next, we examined the influence of estrogen on subcellular localization of phospho-PKM2 [Ser37] in TSC2-null 621–101 cells. The phospho-PKM2 [Ser37] was detected clearly as fluorescent puncta in the nucleus after 30 min estrogen treatment, relative to vehicle control (**Fig 3A, I-II**). Phospho-PKM2 [Ser37] puncta in nuclei were still found after estrogen treatment for 24 hours (**Fig 3A, III**), indicating its stability in nuclei in response to estrogen. Importantly, the estrogen-induced nuclear localization of phospho-PKM2 [Ser37] was abrogated or reduced upon treatment with the potent and selective inhibitor of the estrogen receptor Faslodex (**Fig 3A, IV**), or MAPK inhibitor PD98059 (**Fig 3A, V**). These results suggest that the phosphorylation of PKM2 at Ser37 and its nuclear translocation are estrogen-dependent in 621–101 cells.

Next, we treated TSC2-null 621–101 cells with 10 nM E_2_, E_2_ plus 10 μM Faslodex, E_2_ plus PD98059, or vehicle for 30 minutes or 24 hours, harvested cells, and performed subcellular fractionation. Immunoblot analysis showed that E_2_ treatment for 30 minutes and 24 hours apparently induced the nuclear localization of phospho-PKM2 [Ser37] (**Fig 3C**). Concomitantly, E_2_ treatment for 30 minutes and 24 hours decreased cytoplasmic localization of phospho-PKM2 [Ser37], consistent with findings of immunofluorescent staining. Importantly, Faslodex or PD98059 treatment markedly reduced E_2_-induced nuclear localization of phospho-PKM2. Together, our data using two independent methods demonstrate that E_2_ promotes nuclear translocation of phospho-PKM2 [Ser37] in part via MAPK pathway in TSC2-null cells.

To examine the effect of TSC2 in subcellular localization of phospho-PKM2 [Ser37], we performed immunofluorescent staining and subcellular fractionation in 621–103 cells. E_2_ treatment for 15 minutes and 24 hours moderately increased nuclear localization of phospho-PKM2 [Ser37] (**Fig 3B, I-III**). Moreover, immunoblot analysis of cellular fractions showed that levels of phospho-PKM2 [Ser37] were 85% lower in NUPL1-positive nuclear fraction of TSC2-corrected 621–103 cells relative to that of TSC2-null 621–101 cells (**Fig 3D**). Cytoplasmic levels of phospho-PKM2 were also lower by 36% in TSC2-corrected 621–103 cells relative to that of TSC2-null 621–101 cells. With this additional data regarding TSC2-addback line 621–103, our results strongly suggest that TSC2 negatively regulates E_2_-induced subcellular localization of phospho-PKM2 [Ser37].

### TSC2 negatively regulates PKM2 expression in rapamycin-insensitive manner

**Fig 4:**
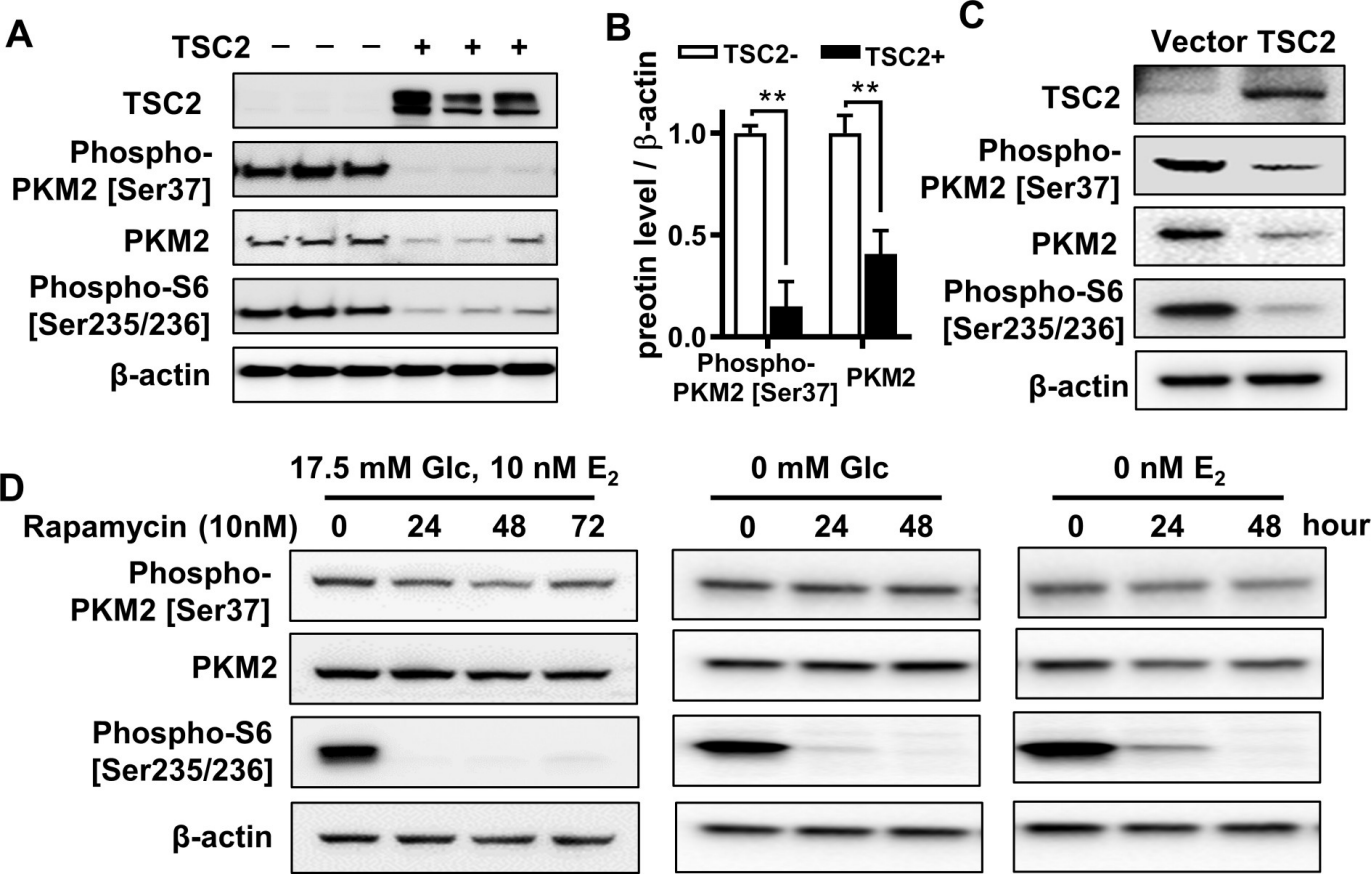
TSC2 regulates PKM2 phosphorylation in an mTORC1-independent manner. **(A)** Immunoblot analysis of TSC2, phospho-PKM2 [Ser37], PKM2 and Phospho-S6 [Ser235/236] in 621–101 (TSC2-) and 621–103 (TSC2+) cells (n = 3); β-actin as a loading control. **(B)** Densitometry analysis of phospho-PKM2 [Ser37] was performed (n = 3). Data are represented as mean ± SEM, **p<0.01, two-sided Student’s t-test. **(C)** 621–101 (TSC2-) cells were transiently electroporated with wild-type TSC2 pcDNA3.1+TSC2 or empty vector pcDNA3.1+, followed by immunoblot analysis of TSC2, phospho-PKM2 [Ser37], PKM2 and Phospho-S6 [Ser235/236] were performed. **(D)** Immunoblot analysis of TSC2, phospho-PKM2 [Ser37], PKM2 and Phospho-S6 [Ser235/236] in 621–101 cells treated with rapamycin (10 nM) for 0, 24, 48, and 72 hours in the culture medium containing 17.5 mM Glc and 10 nM E_2_ (left panel), or the Glc deprivation medium (middle panel) and E_2_ deprivation medium (right panel).

To determine how PKM2 expression is regulated, we first investigated the effect of TSC2 gene expression on the protein level and phosphorylation of PKM2. We found that TSC2-reexpression markedly decreased PKM2 protein levels by 59% and PKM2 phosphorylation [Ser37] by 85% in 621–101 cells (**Fig 4A and 4B**). However, to address a related authentication issue pertaining the potential clonal variation in 621–101 isogenic lines, we transiently transfected 621–101 cells with wild-type TSC2 (pcDNA3.1+TSC2) and empty vector pcDNA3.1+. Immunoblot analysis showed that TSC2 overexpression decreased S6 phosphorylation, **(Fig 4C**), as expected. Importantly, TSC2 overexpression markedly reduced levels of PKM2 phosphorylation by 64% and PKM2 protein by 76% relative to vector control, respectively **(Fig 4C)**. Together, our data indicate that TSC2 negatively regulates PKM2 phosphorylation.

To further investigate whether the specific regulation of PKM2 in TSC2-deficient cells depends on mTORC1 activity, the mTORC1 inhibitor rapamycin was used to treat 621–101 cells lacking TSC2, at indicated time points, followed by immunoblotting. Rapamycin treatment did not affect the protein levels or Ser37 phosphorylation of PKM2, whereas phosphorylation of S6 [Ser235/236] was strongly suppressed (**Fig 4D, left panel**), as expected. To determine whether glucose availability affects the sensitivity of PKM2 phosphorylation to rapamycin, we cultured 621–101 cells in glucose-free (Glc 0 mM) or E_2_-free conditions. In addition, cells were treated with 10 nM rapamycin for 24 and 48 hours. Rapamycin treatment potently reduced S6 phosphorylation, as expected. However, rapamycin did not affect PKM2 phosphorylation (**Fig 4D, middle and right panel**). Together, our data indicate that PKM2 phosphorylation is insensitive to rapamycin treatment in either glucose-rich or glucose-free conditions.

### PKM2 activation is independent of mTOR in TSC2-null cells

**Fig 5:**
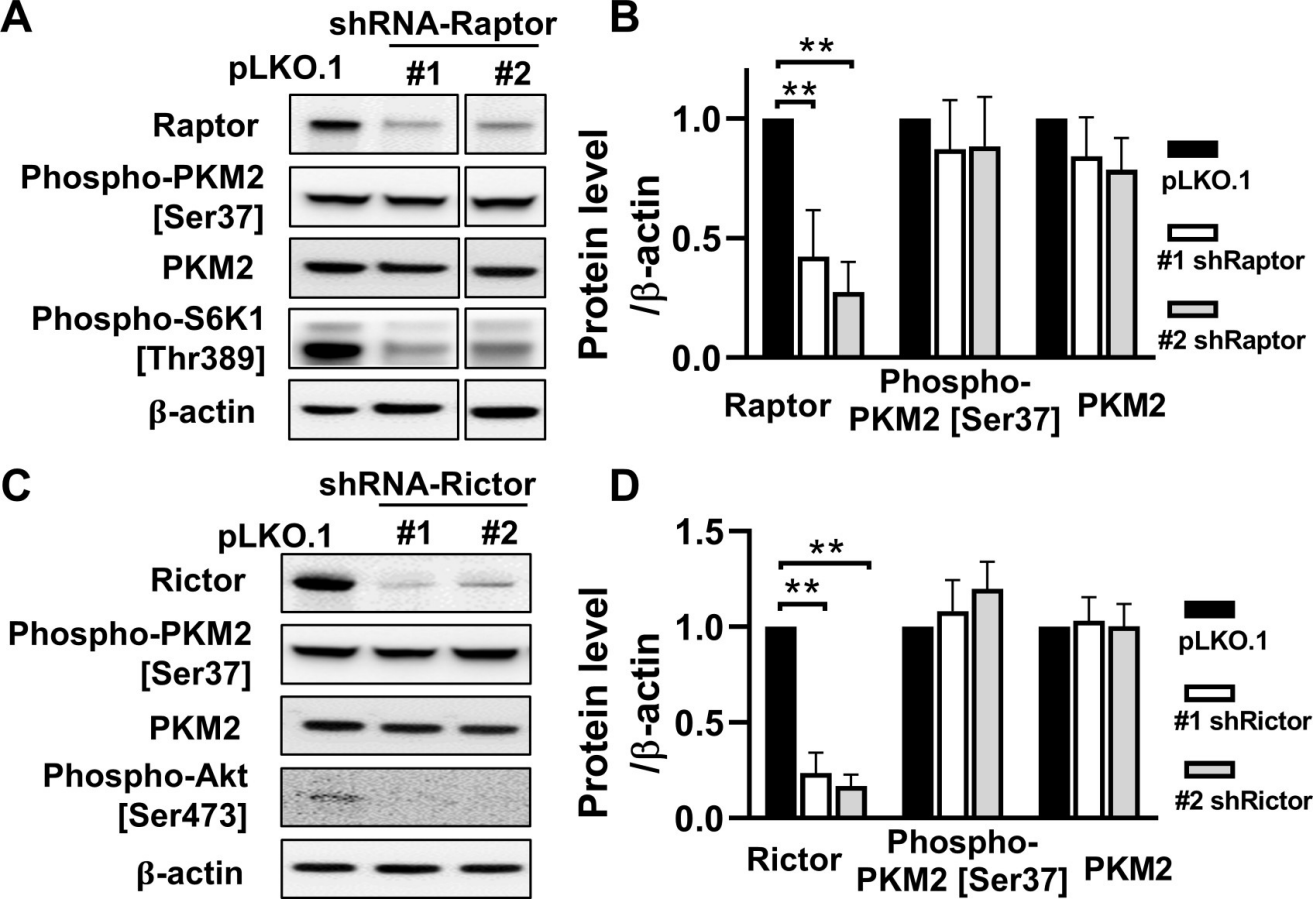
Selective interference of mTORC1/RAPTOR or mTORC2/Rictor doesn’t alter PKM2 expression. **(A)** 621–101 cells were infected with lentiviral particles of shRNA-Raptor (#1 and #2) targeting different regions within the same gene or of empty vector pLKO.1. Immunoblot analysis of Raptor, phospho-PKM2 [Ser37], PKM2 and Phospho-S6K1 [Thr389]; β-actin as a loading control. **(B)** Densitometry analysis of Raptor, phospho-PKM2 [Ser37] and PKM2 from repeating the whole experiment independently three times (n = 3). **(C)** 621–101 cells were infected with lentiviral particles of shRNA-Rictor (#1 and #2) targeting different regions within the same gene or of empty vector pLKO.1. Immunoblot analysis of Rictor, phospho-PKM2 [Ser37], PKM2 and Phospho-Akt [Ser473]; β-actin as a loading control. **(D)** Densitometry analysis of Rictor, phospho-PKM2 [Ser37] and PKM2 from repeating the whole experiment independently three times (n = 3). Data are represented as mean ± SEM, **p<0.01, two-sided Student’s t-test.

To determine whether mTORC1 or mTORC2 specifically regulates PKM2 expression, we used two independent shRNAs to deplete Raptor or Rictor, respectively. As expected, knockdown of Raptor by 58% (#1 shRaptor) and 73% (#2 shRaptor) led to decreased levels of phospho-S6K1 [Thr389], a direct target of mTORC1 (**Fig 5A and 5B**). Knockdown of Rictor by 76% (#1 shRictor) and 83% (#2 shRictor) lowered levels of phospho-Akt [Ser473], a direct target of mTORC2 (**Fig 5C and 5D**), demonstrating their knockdown efficiency. However, neither Raptor knockdown nor Rictor knockdown altered the protein levels of phospho-PKM2 [Ser37] or PKM2. Collectively, our data reveal that TSC2 negatively regulates PKM2 expression in an mTORC1- and mTORC2-independent manner.

### Accumulation of phospho-PKM2 is evident in pulmonary LAM nodules from TSC/LAM patients

**Fig 6:**
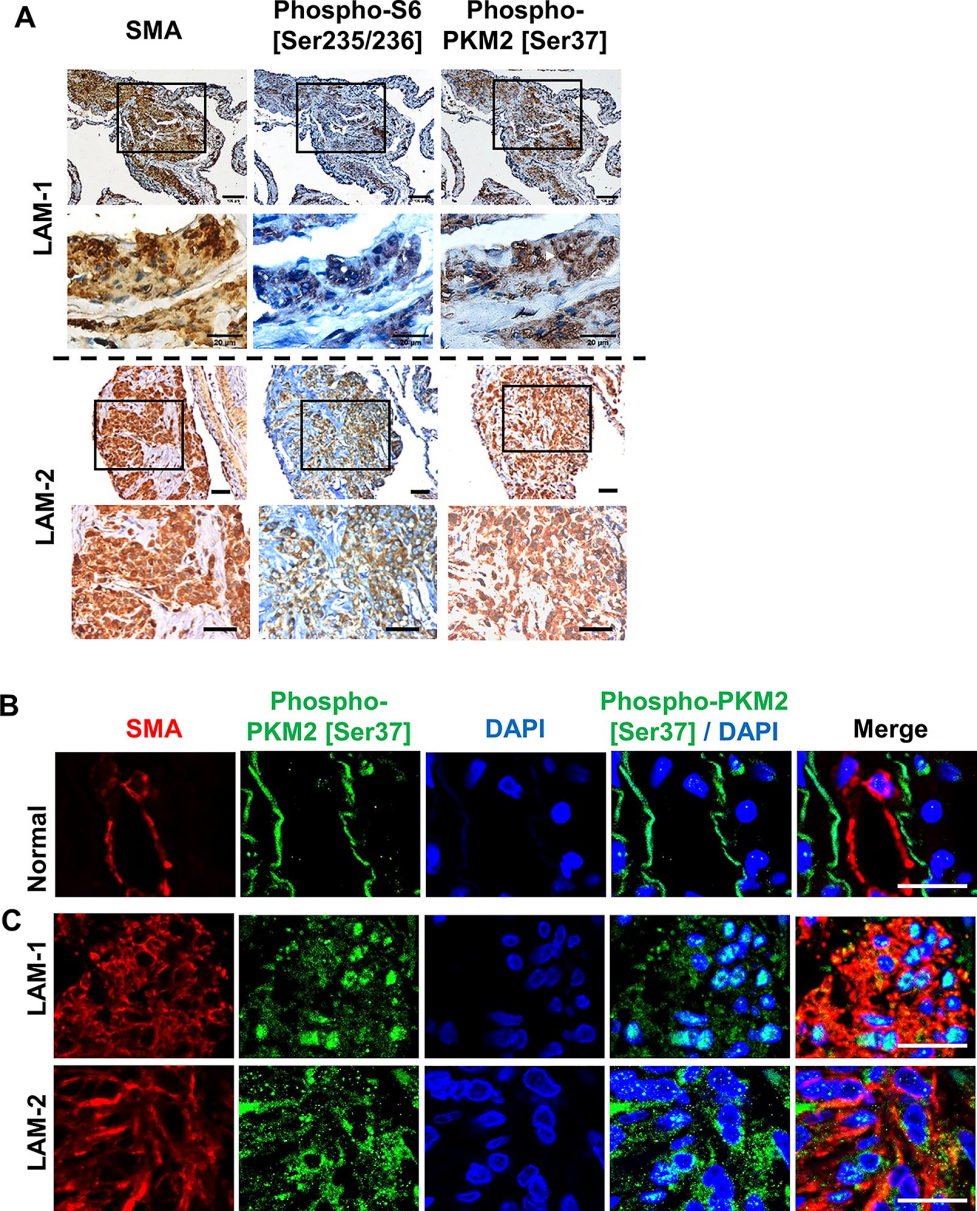
Accumulation of phospho-PKM2 [Ser37] is evident in pulmonary LAM nodules. Immunohistochemical staining of SMA, phospho-S6 [Ser235/236] and phospho-PKM2 [Ser37] in **(A)** pulmonary LAM lungs from two LAM subjects (LAM-1 and LAM-2). Scale bar represents 100 μm. Immunofluorescent co-staining of phospho-PKM2 [Ser37] and SMA in (**B**) normal lung tissue, (**C)** and two cases of pulmonary LAM lungs (LAM-1 and LAM-2). Nuclei were stained with DAPI. Scale bar represents 20 μm.

To determine the clinical relevance of phospho-PKM2, we assessed the abundance of phospho-PKM2 [Ser37] using immunohistochemical staining in two cases of pulmonary LAM lungs. Phospho-PKM2 [Ser37] accumulation was prominent in pulmonary LAM nodule cells that were positive for smooth muscle actin (SMA) and phospho-S6 [Ser235/236] (**Fig 6A**). Low levels of phospho-PKM2 were observed in SMA-positive bronchial smooth muscle cells in normal lung (**Fig 6B**). In contrast, immunofluorescent confocal microscopy showed that pulmonary LAM lesion cells accumulated both nuclear and cytoplasmic phospho-PKM2 in the two LAM lungs (**Fig 6C**). These data indicate that phosphorylation of PKM2 is likely associated with specific tumor growth in LAM lesions.

### Expression of ERα and ERβ is evident in TSC2-null cells

**Fig 7:**
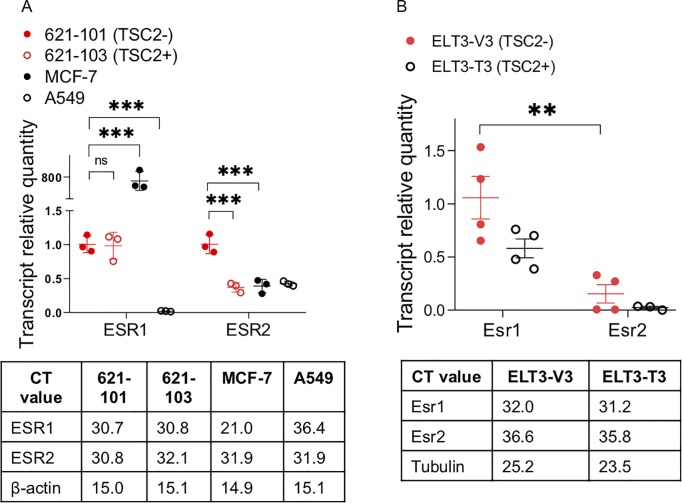
Expression of ERα and ERβ is evident in LAM-derived 621–101 and rat-derived ELT3 cells. The relative transcript levels of ERα and ERβ in **(A)** LAM patient-derived cells 621–101 (TSC2-) and 621–103 (TSC2+), **(B)** rat-derived cells ELT3-V3 (TSC2-) and ELT3-T3 (TSC2+), breast cancer MCF-7 cells, and lung adenocarcinoma A549 cells. The actual mean CT values (n = 3-4/cell type) of the ERα and ERβ transcript were shown in the tables. ** p < 0.01, *** p < 0.001, Student *t* test.

Studies have shown that LAM patient-derived 621–101 cells and rat uterine leiomyoma-derived ELT3 cells express ERα and respond to estrogen stimulation [20, 23, 25, 26]. To examine the status of the expression of ERα and ERβ expression in these cell models, we measured their transcript levels using quantitative real-time RT-PCR. The relative transcript level of ERα (ESR1) was significantly higher in 621–101 cells (CT = 30.7) relative to lung adenocarcinoma A549 cells (CT = 36.4) (p < 0.001; **Fig 7A**), although TSC2-reexpression (621–103 cells) did not affect ESR1 expression. However, the transcript level of ESR1 was much lower in 621–101 cells than that in breast cancer MCF-7 cells, similar to previous findings [23]. Importantly, the transcript level of ERβ (ESR2) was higher in 621–101 cells (CT = 30.8) relative to 621–103, MCF-7 and A549 cells (CT ~ 32) (Fig 7A). Moreover, the transcript level of ERβ (Esr2) (CT = 36.6) was much lower than ERα (Esr1) (CT = 32.0) in Tsc2-null ELT3-V3 cells, although TSC2-reexpression did not affect the expression of Esr1 or Esr2 in ELT3-T3 cells (**Fig 7B**).

## Discussion

LAM is a devastating lung disease affecting young women that is characterized by metastasis of smooth muscle cells to the lungs and emphysema-like destruction of the lung parenchyma [27–30]. LAM is associated with *TSC2* mutations [31] resulting in activation of the mechanistic target of rapamycin complex 1 (mTORC1) [32], A seminal clinical trial showed that the mTORC1 inhibitor sirolimus stabilizes lung function and improves symptoms in LAM patients while drug exposure continues [33], but long-term benefit and toxicity are unknown, and some patients do not respond. This limitation of sirolimus has engendered multiple efforts to attempt to develop better, or at least complementary, treatment approaches. Despite many advances in our understanding of the importance of mTORC1-dependent and -independent signaling pathways that are central to LAM pathogenesis, the underlying basis of the sexual dimorphism for symptomatic LAM is essentially unknown. In this study, we show evidence that this may at least in part be mediated by estrogen regulation of PKM2. We show that estrogen treatment increases the phosphorylation of PKM2 at Serine 37 and induces the nuclear translocation of phospho-PKM2; treatment with the estrogen receptor antagonist Faslodex blocks these actions. Morevover, accumulation of phosphorylated PKM2 was increased in pulmonary nodule cells from TSC/LAM patients.

Faslodex disrupts ligand binding, receptor dimerization and nuclear translocation, and degradation of both ERα and ERβ [34, 35]. Studies including ours have shown the expression of both ERα and ERβ in LAM cells [26, 36], suggesting the interplay between ERα and ERβ in LAM progression. Opposing effects of estrogen receptor subtypes ERα and ERβ have been implicated in breast cancer cells [37]. ERα plays a pro-proliferative role and ERβ can exert an anti-proliferative action by controlling cell-cycle regulators in most cell types, depending on the differential expression of ER subtypes [38, 39]. In our cell-based models, the transcript levels of ERα were higher than that of ERβ in ELT3-V3 cells, but comparable in 621–101 and (Fig 7). Our previous studies have demonstrated that Faslodex inhibits the estrogen-promoted lung metastasis of ELT3 cells in vivo [40], although the specific role of ERα or ERβ was not addressed. In the present study, we observed that Faslodex treatment decreased estrogen-induced phosphorylation of PKM2 and nuclear translocation of phospho-PKM2 (Fig 3A, IV). We speculate that both ERα and ERβ mediate estrogen actions on PKM2 phosphorylation and proliferation of TSC2-null cells. Because agonists and antagonists to both ERα and ERβ are available, it would be of paricular interest to assess the specific impact of ER subtypes on celluar functions, which have not been explored in LAM. We postulate that ERβ-specific agonist would decrease, and ERβ-specific antagonists would increase proliferation and PKM2 activation in LAM cells, which could be addressed in future studies.

Alterations in cellular energy metabolism are a hallmark of cancer [41], and metabolic reprogramming is particularly critical for the survival of Tsc2-deficient tumor cells [14, 42–44]. TSC2 deficiency leads to increased transcription of glucose metabolism genes [9] and the cells with mTORC1 activation express high levels of the glycolytic protein PKM2 [10]. The glucose dependent survival of TSC2-deficient cells has been reported [43], suggesting that glucose metabolism is essential for the growth of TSC2-deficient cells. However, the mTORC1 inhibitor, rapamycin (or sirolimus) reduces lactate production but does not affect cellular ATP levels in

*Tsc2*^-/-^ MEF cells [44]. It has been reported that TSC2-deficient cells exhibit autophagy-dependent alteration of glucose metabolism rewiring to pentose phosphate pathway [42]. Together, these reports highlight connections between cellular metabolic alterations and glucose utilization that likely impact the survival of TSC2-deficient cells.

It has been demonstrated that epidermal growth factor receptor (EGFR)-activated ERK2 phosphorylates PKM2 at Serine 37, thereby promoting nuclear translocation of PKM2 [12]. Previous studies including ours have shown that estrogen increases ERK1/2 phosphorylation in TSC2-deficient LAM-derived cells [14, 18, 26] and rat uterine leiomyoma-derived ELT3 cells [15–17, 19, 30, 45, 46]. We have previously shown that estrogen promotes the lung metastasis of Tsc2-deficient ELT3 tumors in an MEK1/2-ERK1/2-dependent manner [17]. Moreover, TSC2 appears to negatively regulate the expression of PKM2 in an mTORC1- and mTORC2-independent manner, although studies have demonstrated that mTORC1 and mTORC2 are required for proliferation and survival of TSC2-null LAM-derived cells [32, 47].

Importantly, our study shows that phosphorylated PKM2 is evident in the nucleus of LAM patient-derived cells *in vitro* and in pulmonary LAM nodule cells *in vivo*. Phosphorylated PKM2 [S37] and its nuclear translocation promote the Warburg effect and tumorigenesis [12]. Monomeric PKM2 translocates into the nucleus, where it functions as a transcriptional co-activator of β-catenin and upregulates the expression of c-Myc and cyclin D1 [48], thereby promoting the Warburg effect and cell cycle progression, respectively [13]. These findings will warrant future investigation of the important role of PKM2 in estrogen-driven LAM progression.

LAM can lead to respiratory failure and death [49–51]. The Multicenter International LAM Efficacy of Sirolimus Trial (MILES Trial) demonstrated that the mTORC1 inhibitor sirolimus (rapamycin) stabilizes lung function and improves the symptoms in women with LAM. However, lung function decline resumed upon drug cessation [33]. Therefore, despite advances in the clinical care of women with LAM, there remains a critical need for improved therapeutic options. PKM2 expression can only be suppressed by TSC2 reconstitution and is not significantly affected by the mTORC1 inhibitor rapamycin, or Raptor depletion using shRNA, suggesting that TSC2 deficiency upstream of the mTORC1 pathway is the leading cause of PKM2 upregulation. Interestingly, we also showed that knockdown of mTORC2 component Rictor does not affect the protein of levels or phosphorylation of PKM2, indicative of mTORC2-indepent regulation of PKM2 expression and activation in TSC2-deficient cells. In this study, our data suggest that PKM2 upregulation is likely a direct consequence of TSC2 loss.

Collectively, our data suggest that inhibiting estrogen-dependent cellular metabolic pathways could block the pro-survival effects of estrogen on LAM cells without need to ablate the entire hormonal signaling axis. Hormonal ablation therapy for breast cancer patients decreases circulating hormone levels and increases the development of osteoporosis and bone fracture [52, 53]. Thus, in the long term, it is possible that, compared to hormonal ablation, metabolically-focused strategies in LAM could have preferable side effect profiles with regard to bone health and biochemical parameters including serum calcium, serum phosphorus and bone specific isoform of alkaline phosphatase [54]. Recent studies have demonstrated therapeutic potentials of targeting dysregulated cellular metabolic pathways including glucose metabolism and autophagy addication using hydroxychloroquine or resveratrol in LAM [25, 55–58]. However, additional pre-clinical and clinical studies will be needed to test this concept.

## Supporting information

S1 Fig Original blot/gel image data Fig 1E. **Estrogen promotes the growth of TSC2-deficient cells via PKM2 in a glucose-dependent manner. (E)** Immunoblot analysis of PKM2 in 621–101 cells infected with lentiviral particles of shRNA-PKM2 (#1 and #2) targeting different regions within the same gene or of empty vector pLKO.1 as control. β-actin as a loading control.
(TIF)

S2 Fig Original blot/gel image data Fig 2A and 2C. Estrogen induces PKM2 phosphorylation. **(A)** 621–101 and 621–103 cells in triplicate after E_2_ (10 nM) treatment for 2 hours. Immunoblot analysis of phospho-PKM2 [Ser37], PKM2 and Phospho-ERK1/2 [Thr202/Tyr204]. **(C)** 621–101 cells were treated with vehicle, E_2_ (10 nM), Faslodex (10 μM), or E_2_ (10 nM) plus Faslodex (10 μM) for 24 hours in glucose-rich (Glc 17.5 mM) or glucose-free medium (Glc 0 nM), followed by immunoblot analysis of phospho-PKM2 [Ser37] and PKM2. β-actin as a loading control.
(TIF)

S3 Fig Original blot/gel image data Fig 3C and 3D. **Estrogen induces nuclear translocation of phospho-PKM2 [S37] in a TSC2-dependent manner. (C)** Immunoblot analysis of phospho-PKM2 [Ser37], NUPL1 and S6 in cytoplasmic and nuclear fractions isolated from 621–101 cells in the same treatment as (A). **(D)** Immunoblot analysis of phospho-PKM2 [Ser37], TSC2, NUPL1 and S6 in cytoplasmic and nuclear fractions isolated from 621–101 (TSC2-) and 621–103 (TSC2+) cells.
(TIF)

S4 Fig Original blot/gel image data Fig 4A, 4C and 4D. **TSC2 regulates PKM2 phosphorylation in an mTORC1-independent manner. (A)** Immunoblot analysis of TSC2, phospho-PKM2 [Ser37], PKM2 and Phospho-S6 [Ser235/236] in 621–101 (TSC2-) and 621–103 (TSC2+) cells (n = 3); β-actin as a loading control. **(C)** 621–101 (TSC2-) cells were transiently electroporated with wild-type TSC2 pcDNA3.1+TSC2 or empty vector pcDNA3.1+, followed by immunoblot analysis of TSC2, phospho-PKM2 [Ser37], PKM2 and Phospho-S6 [Ser235/236] were performed. **(D)** Immunoblot analysis of TSC2, phospho-PKM2 [Ser37], PKM2 and Phospho-S6 [Ser235/236] in 621–101 cells treated with rapamycin (10 nM) for 0, 24, 48, and 72 hours in the culture medium containing 17.5 mM Glc and 10 nM E_2_ (left panel), or the Glc deprivation medium (middle panel) and E_2_ deprivation medium (right panel).
(TIF)

S5 Fig Original blot/gel image data Fig 5A and 5C. **Selective interference of mTORC1/RAPTOR or mTORC2/Rictor doesn’t alter PKM2 expression. (A)** 621–101 cells were infected with lentiviral particles of shRNA-Raptor (#1 and #2) targeting different regions within the same gene or of empty vector pLKO.1. Immunoblot analysis of Raptor, phospho-PKM2 [Ser37], PKM2 and Phospho-S6K1 [Thr389]; β-actin as a loading control. **(C)** 621–101 cells were infected with lentiviral particles of shRNA-Rictor (#1 and #2) targeting different regions within the same gene or of empty vector pLKO.1. Immunoblot analysis of Rictor, phospho-PKM2 [Ser37], PKM2 and Phospho-Akt [Ser473]; β-actin as a loading control.
(TIF)
