# Supplementary figures and images for: Estrogen activates pyruvate kinase M2 and increases the growth of TSC2-deficient cells
Source: PLoS One. 2020 Feb 20;15(2):e0228894. doi: 10.1371/journal.pone.0228894 (PMC7032738; doi:10.1371/journal.pone.0228894)

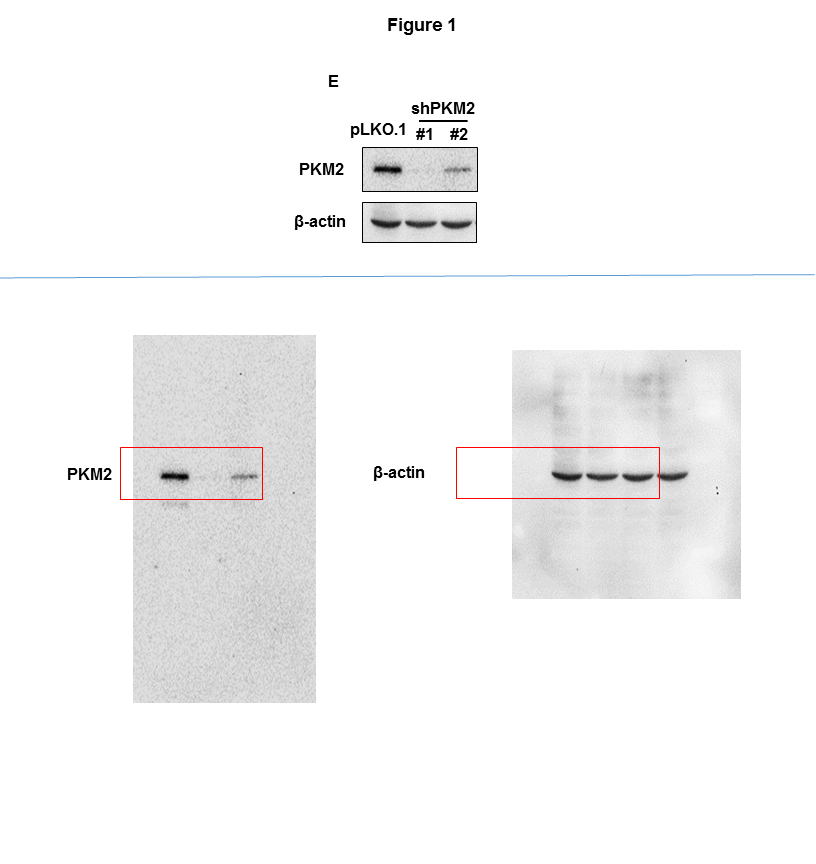

Supplement: S1 Fig — Estrogen promotes the growth of TSC2-deficient cells via PKM2 in a glucose-dependent manner. (E) Immunoblot analysis of PKM2 in 621–101 cells infected with lentiviral particles of shRNA-PKM2 (#1 and #2) targeting different regions within the same gene or of empty vector pLKO.1 as control. β-actin as a loading control. (TIF) [file pone.0228894.s001.tif]

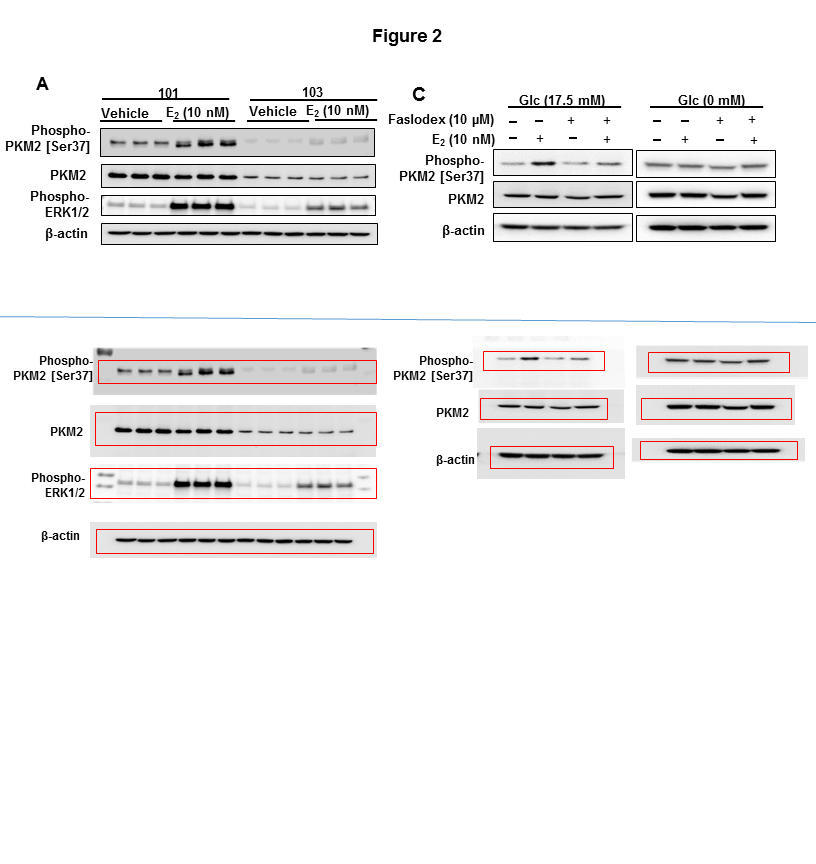

Supplement: S2 Fig — Estrogen induces PKM2 phosphorylation. (A) 621–101 and 621–103 cells in triplicate after E2 (10 nM) treatment for 2 hours. Immunoblot analysis of phospho-PKM2 [Ser37], PKM2 and Phospho-ERK1/2 [Thr202/Tyr204]. (C) 621–101 cells were treated with vehicle, E2 (10 nM), Faslodex (10 μM), or E2 (10 nM) plus Faslodex (10 μM) for 24 hours in glucose-rich (Glc 17.5 mM) or glucose-free medium (Glc 0 nM), followed by immunoblot analysis of phospho-PKM2 [Ser37] and PKM2. β-actin as a loading control. (TIF) [file pone.0228894.s002.tif]

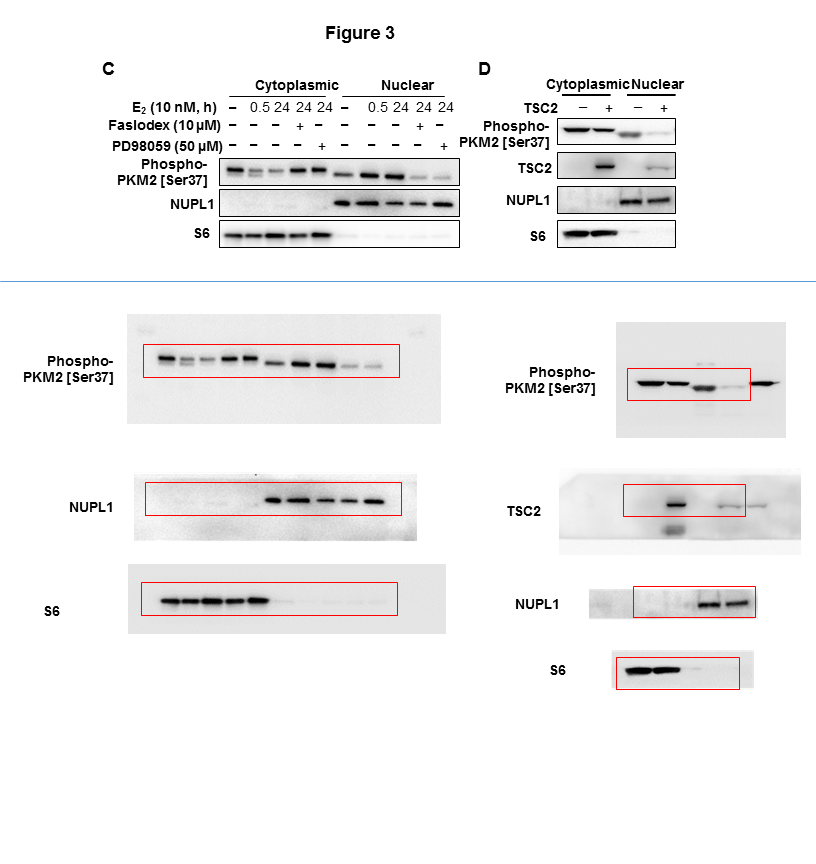

Supplement: S3 Fig — Estrogen induces nuclear translocation of phospho-PKM2 [S37] in a TSC2-dependent manner. (C) Immunoblot analysis of phospho-PKM2 [Ser37], NUPL1 and S6 in cytoplasmic and nuclear fractions isolated from 621–101 cells in the same treatment as (A). (D) Immunoblot analysis of phospho-PKM2 [Ser37], TSC2, NUPL1 and S6 in cytoplasmic and nuclear fractions isolated from 621–101 (TSC2-) and 621–103 (TSC2+) cells. (TIF) [file pone.0228894.s003.tif]

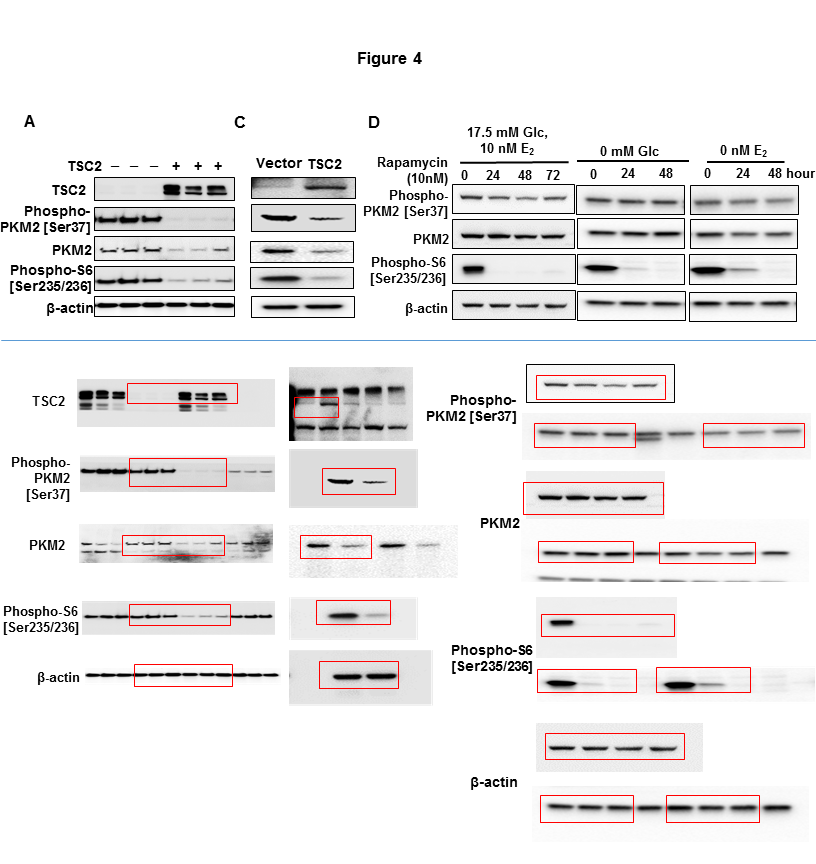

Supplement: S4 Fig — TSC2 regulates PKM2 phosphorylation in an mTORC1-independent manner. (A) Immunoblot analysis of TSC2, phospho-PKM2 [Ser37], PKM2 and Phospho-S6 [Ser235/236] in 621–101 (TSC2-) and 621–103 (TSC2+) cells (n = 3); β-actin as a loading control. (C) 621–101 (TSC2-) cells were transiently electroporated with wild-type TSC2 pcDNA3.1+TSC2 or empty vector pcDNA3.1+, followed by immunoblot analysis of TSC2, phospho-PKM2 [Ser37], PKM2 and Phospho-S6 [Ser235/236] were performed. (D) Immunoblot analysis of TSC2, phospho-PKM2 [Ser37], PKM2 and Phospho-S6 [Ser235/236] in 621–101 cells treated with rapamycin (10 nM) for 0, 24, 48, and 72 hours in the culture medium containing 17.5 mM Glc and 10 nM E2 (left panel), or the Glc deprivation medium (middle panel) and E2 deprivation medium (right panel). (TIF) [file pone.0228894.s004.tif]

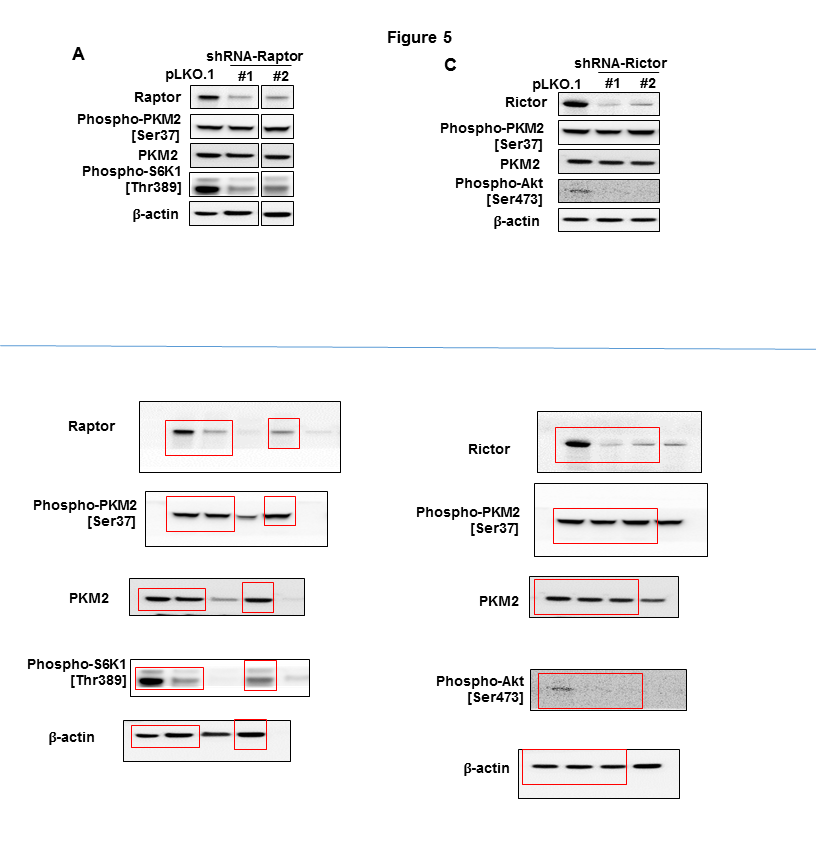

Supplement: S5 Fig — Selective interference of mTORC1/RAPTOR or mTORC2/Rictor doesn’t alter PKM2 expression. (A) 621–101 cells were infected with lentiviral particles of shRNA-Raptor (#1 and #2) targeting different regions within the same gene or of empty vector pLKO.1. Immunoblot analysis of Raptor, phospho-PKM2 [Ser37], PKM2 and Phospho-S6K1 [Thr389]; β-actin as a loading control. (C) 621–101 cells were infected with lentiviral particles of shRNA-Rictor (#1 and #2) targeting different regions within the same gene or of empty vector pLKO.1. Immunoblot analysis of Rictor, phospho-PKM2 [Ser37], PKM2 and Phospho-Akt [Ser473]; β-actin as a loading control. (TIF) [file pone.0228894.s005.tif]
